# Movement Disorders and Psychosis, a Complex Marriage

**DOI:** 10.3389/fpsyt.2014.00190

**Published:** 2015-01-09

**Authors:** Peter N. van Harten, P. Roberto Bakker, Charlotte L. Mentzel, Marina A. Tijssen, Diederik E. Tenback

**Affiliations:** ^1^Psychiatric Centre GGz Centraal, Innova, Amersfoort, Netherlands; ^2^School for Mental Health and Neuroscience, Maastricht University, Maastricht, Netherlands; ^3^Department of Neurology, University Medical Center Groningen, University of Groningen, Groningen, Netherlands

**Keywords:** movement disorders, psychotic disorders, tardive dyskinesia, schizophrenia, instrumental assessment

Most clinicians relate parkinsonism and dyskinesia directly to acute and tardive drug-induced movement disorders. However, parkinsonism and dyskinesia are also present in antipsychotic-naïve patients with psychotic disorders. In this paper, we want to highlight the clinical value of these spontaneous movement disorders and want to discuss the concept of “non-mental signs.”

## Acute Drug-Induced Movement Disorders

Acute drug-induced movement disorders, such as acute dystonia, parkinsonism, and akathisia, are very common side effects of dopamine blocking agents. A causal relationship between these movement disorders and antipsychotics is beyond any doubt if (i) antipsychotic-naïve psychotic patients without movement disorders receive antipsychotics and develop these side effects, (ii) they disappear after dose reduction or cessation of the antipsychotics, and (iii) this on–off mechanism can be repeated.

## Tardive Syndromes

The relationship between tardive syndromes and antipsychotics is far more complex because they start after months to years of treatment with antipsychotics and can also be suppressed by antipsychotics. Tardive suggests drug induced, and also spontaneous hyperkinetic dyskinesias, such as “grimacing” and “irregular movements of tongue and lips” (and also parkinsonism), are prevalent in antipsychotic-naïve psychotic patients and have been described by Kraepelin and Bleuler more than a 100 years ago (1).

In patients with long-term use of antipsychotics, there is no test to differentiate between drug-induced tardive and spontaneous movement disorders. The prevalence of drug-induced tardive dyskinesia is substantial and increases with age, the same counts for spontaneous movement disorders such as dyskinesia, bradykinesia, and soft neurological signs related to schizophrenia (2–15). Also, a meta-analysis showed that in antipsychotic-naïve patients with schizophrenia the risk of dyskinesia and parkinsonism are three and five times higher than in healthy controls, respectively (16). Furthermore, another study in antipsychotic-naïve patients showed a prevalence of dyskinesia and parkinsonism of 13 and 18%, respectively, with the use of clinical rating scales, which increased to 20 and 28%, respectively, with the use of instrumental assessment (17).

On the other hand, several findings suggest a direct relationship between antipsychotics and tardive dyskinesia. First, non-psychiatric patients may also develop tardive dyskinesia after long-term use of dopamine blocking agents, e.g., long-term use of metoclopramide to treat nausea, or antipsychotics for insomnia (18, 19). Furthermore, in older patients receiving first-generation antipsychotics for the first time the yearly incidence of tardive dyskinesia is extremely high, over 20%, which is much higher than the incidence of spontaneous dyskinetic movement in older patients (12, 13). Also, tardive dyskinesia may disappear after cessation of antipsychotics or after a switch to clozapine. These findings suggest a direct relationship between antipsychotics and tardive dyskinesia.

Based on the studies mentioned above, it is clear that the assumption that antipsy- chotics are responsible for tardive dyskinesia is at least incomplete. Indeed, movement disorders can be considered an intrinsic feature of the disease process and implicate dysfunction in cortical–basal ganglia-cortical circuitry (11). The role of the antipsychotics may be modification of the disease-based motor disorder and anti- psychotics can both improve and unmask primary motor abnormalities (10).

The clinical importance of spontaneous movement disorders is also emphasized by the relationship between spontaneous parkinsonism and cognitive dysfunction. In a prospective study in antipsychotic-naïve patients with first-episode psychosis, spontaneous parkinsonism at baseline showed high 6-month predictive values for cognitive impairment (9).

## Pathophysiology

The pathogenesis of tardive dyskinesia remains unresolved. Several hypotheses have been proposed such as dopamine 2 (D2)-receptor hypersensitivity, striatal neurodegeneration, maladaptive synaptic plasticity, and enhanced serotonin 2 (5-HT2)-receptor signaling and recently up regulation of striatal D3 receptors had been suggested in a primate model (20). Although none of these models have been confirmed sufficiently they have in common the disturbance of the balance in the motor circuit of the basal ganglia in which dopamine plays a central role. The dopamine (and possibly also the acetylcholine) dysregulation in the basal ganglia-thalamo-cortical loops may result in hyper or hypokinetic movements whereas dopamine dysregulation in other brain areas may result in the development of psychosis (21).

Another model is based on synaptic dysregulations in which the core hypothesis is that non-functional astrocytic receptors may cause an unconstrained synaptic information flux, such that glia lose their modulatory function in glial–neuronal interaction (tripartite synapses) (22). Dysregulation of tripartite synapses would occur with dopamine synapses throughout the brain and may be related to both motoric and mental symptoms.

## Clinical Relevance

The clinical relevance for measuring dyskinesia and/or parkinsonism in first-episode psychotic disorders is based on several follow-up studies showing that they predict poor prognosis, increased cognitive impairment, poorer response to antipsychotics, and an increased risk for drug-induced movement disorders (9, 11, 23).

Also, in individuals at ultra-high risk for psychosis (UHR group) the assessment of spontaneous movement disorders may be highly relevant. Several studies suggest that subtle abnormal movements are predictive for conversion to psychosis later. The current screening strategy focuses on mental symptoms and has a limited conversion rate to psychosis, around 20–40%, giving to many false positives. It could be that adding measurement of movement disorders to the screening strategy will reduce the number of false positives. Indeed, studies show (i) more abnormal movements in the UHR group than in the control group, (ii) a relationship between the severity of the abnormal movements and the severity of prodromal signs (positive, negative, and total) at baseline, (iii) a relationship between an increase in severity of the abnormal movements with an increase of prodromal signs during follow-up, and (iv) a higher risk to convert to psychosis at follow-up in the UHR groups with abnormal movements at baseline than those without (24, 25).

Detection of those in the UHR group who will convert to psychosis is relevant as a meta-analysis showed the effectiveness of some interventions to prevent or postpone a first-episode of psychosis (26).

## Relationship between Movement, Cognitive, and Emotional Disorders

Obeso et al. describe that the basal ganglia are intimately connected with the cortex through several segregated but parallel loops. These loops are subdivided into motor, associative (cognitive), and limbic (emotional) domains and are related to the control of movement, behavior and cognition, and reward and emotions, respectively. When one or more of these circuits become dysfunctional they can generate movement disorders, behavioral, cognitive abnormalities, or mood changes. They suggest, for example that the combination of nigrostriatal denervation and dopaminergic drugs, as seen in Parkinson’s disease, may induce behavioral disorders such as impulse control disorders and that this may be the behavioral counterpart of hyperkinetic disorders such as dyskinesia (27). Similar with this idea is the concept that dysregulation of dopaminergic activity in dopaminergic related brain areas lead to positive and negative symptoms in psychotic disorders and that these symptoms are the behavioral counterpart of dyskinesia and bradykinesia, respectively. It has been suggested that psychotic patients with abnormal movements, compared to those without, have a more severely dysregulated dopamine system (28). This may explain the clustering of abnormal movements with cognitive and negative symptoms and the relationship with poor prognosis. Also, a correlation has been found between tardive dyskinesia and cognitive symptoms (29). It could be that drug-induced movement disorders are related to a more vulnerable dopamine system and subsequently to an increased risk for dyskinesia and negative and cognitive symptoms. In line with the vulnerability concept is the relationship found between early extrapyramidal symptoms such as parkinsonism and an increased risk for developing tardive dyskinesia in the future (30, 31). However, the underlying dysfunction(s) that provoke(s) spontaneous movement abnormalities, tardive dyskinesia, cognitive impairment, negative symptoms, and emotional disturbances remains unclear. It is unlikely that one neurotransmitter, i.e., dopamine is responsible. Although, dysfunction of the modulatory activity of dopamine plays an important role in the clinical manifestations mentioned above, also acetylcholine, which is released across the entire striatal network by striatal cholinergic interneurons, has neuromodulatory properties in the basal ganglia. Furthermore, other neurotransmitters are involved, such as glutamatergic inputs from the cerebral cortex and thalamus to striatal spiny projection neurons (21).

## Non-Mental Signs

Based on the presence of motor, associative (cognitive), and limbic (emotional) loops in the basal ganglia, we want to introduce the concept of non-mental signs (dyskinesia and parkinsonism) in psychotic disorders. This concept is the equivalent of non-motor signs (mood disorders, apathy, anxiety, etc.) in Parkinson’s disease (32). The severity of non-mental signs may have a direct relationship with the severity of dysregulation of the dopamine system. An advantage of non-mental signs is the possibility to measure them objectively and several research groups have developed instruments to measure these non-mental signs instrumentally. Instrumental assessment of movement disorders is sensitive, valid, and reliable and a motor test battery that will quantify the main motor functions has been suggested (33–38). In addition, instrumental measurement can also detect subclinical movement abnormalities and these assessments may be used to predict the course of a (pre)psychotic disorder and can be used to develop preventive strategies.

In conclusion, we suggest classifying movement disorders in psychotic disorders or in UHR groups as non-mental signs. Instrumental measurements of these non-mental signs are objective and have clinical implications for prognosis, diagnosis, and treatment of psychotic disorders. In UHR groups adding non-mental signs to the screening strategy may reduce the number of false positives. Non-mental signs could become one of the first biomarkers in psychiatric screening programs.

## Conflict of Interest Statement

The authors declare that the research was conducted in the absence of any commercial or financial relationships that could be construed as a potential conflict of interest.
